# Transforming Pediatric Care Through AI: Bridging the Digital Divide in Health Informatics

**DOI:** 10.2196/82108

**Published:** 2026-05-13

**Authors:** Mercy Mbogori-Kairichi, R Sterling Snead, Julia Marczika, Radha Ambalavanan, Gideon Towett, Alex Malioukis

**Affiliations:** 1 Research Department, The Self Research Institute, 18452 E 111th St, Broken Arrow, OK, 74011, United States, 1 701155273

**Keywords:** health informatics, digital health, pediatrics, artificial intelligence, health information systems, electronic health records

## Abstract

Health informatics and artificial intelligence (AI) technologies are increasingly influencing pediatric health care delivery across diverse health system contexts. These technologies offer opportunities to improve diagnostic accuracy, personalized treatment approaches, and access to care globally. This viewpoint examines how health and public health informatics frameworks, when integrated with AI technologies, may help address persistent challenges in global pediatric care delivery. This paper is a viewpoint informed by selected published studies and international digital health guidance rather than a systematic review. Evidence from clinical implementations suggests that AI applications embedded in standardized electronic health records can facilitate improved pediatric diagnostic processes. For instance, machine learning–based algorithms to diagnose serious bacterial infections among febrile infants have shown high diagnostic accuracy and reduced unnecessary invasive procedures in certain clinical contexts. Case studies from the Pediatric Emergency Care Applied Research Network decision rules, neonatal intensive care units, and autism screening programs reflect diverse applications of AI-enabled clinical decision support across pediatric settings. However, there are concerns regarding implementation due to limitations in interoperability of health information systems, gaps in data standardization, inadequate digital infrastructure in resource-limited settings, and issues related to algorithmic bias and equitable access. We argue that strategic development of interoperable health information systems, standardized data governance frameworks, and equitable digital infrastructure is essential to responsibly realize the potential of AI-enhanced pediatric care at scale.

## Introduction

Artificial intelligence (AI) is increasingly being integrated into health care delivery and digital health systems, contributing to the modernization of medical practice [1]. Health informatics provides the foundational infrastructure and standardized data models that enable AI algorithms to function effectively in real-world settings. The COVID-19 pandemic has increased the use of digital health worldwide, accelerating the integration of AI into pediatric care systems [2]. Emerging evidence suggests promising applications in pediatric settings. For example, machine learning (ML) algorithms applied to clinical data from febrile infants have demonstrated high diagnostic performance, achieving 98.6% sensitivity and 74.9% specificity in identifying serious bacterial infections. The random forest model showed superior diagnostic accuracy (area under the receiver operating characteristic curve: 0.96) and was estimated to potentially reduce 68.5% of unnecessary lumbar punctures [3]. These findings suggest that AI-driven diagnostic models, when supported by robust health informatics infrastructures and structured electronic health record (EHR) environments, can enhance pediatric diagnostic accuracy while reducing unnecessary interventions.

Despite some progress, pediatric health informatics has lagged behind adult-focused digital health advancements. Researchers have raised concerns about the validity and reliability of applying adult-trained AI models to pediatric populations, with potential implications for care quality [4]. In low- and middle-income countries (LMICs), the synergy between AI and informatics may provide a valuable platform for enhancing access and quality in pediatric care. According to the World Health Organization (WHO), under-five mortality remains a major global health challenge, with millions of deaths occurring annually from largely preventable causes [5]. In this context, AI-enabled decision support systems have the potential to facilitate early diagnosis and improved management pathways, although real-world impact remains uneven. However, substantial barriers to adoption persist, including interoperability challenges, data standardization gaps, digital infrastructure limitations, and equity concerns in resource-constrained settings. Much of the current literature examines AI applications and health informatics infrastructure separately, with comparatively limited attention to their integrated implementation within pediatric care settings, especially in relation to equity and real-world challenges. This gap is critical because pediatric populations have unique physiological, developmental, and care delivery characteristics that require specialized informatics frameworks and AI models distinct from adult-focused approaches.

This viewpoint examines how public health informatics frameworks, when integrated with AI technologies such as ML, natural language processing (NLP), and predictive analytics, can support efforts to improve pediatric care delivery across diverse health system contexts. We review successful applications of health informatics and AI in pediatric settings, identify persistent challenges hindering integration, and discuss considerations for implementing informatics frameworks that responsibly leverage AI to support pediatric care. This analysis is intended for pediatric health care providers, health informaticists, policymakers, and researchers working to advance digital health solutions in pediatric care.

## Evidence Informing This Viewpoint

This viewpoint is informed by a purposive selection of published literature and international guidance reports. We conducted a targeted search of PubMed and Google Scholar using key terms related to pediatrics, AI, health informatics, and digital health. We also used gray literature sources, including policy and guidance reports from the WHO and World Bank, to identify relevant evidence published between 2014 and 2025. Search concepts included combinations of terms, such as pediatrics, children, AI, ML, health informatics, digital health, EHRs, clinical decision support, telemedicine, implementation, and health equity. Using the selected literature, we identified 3 illustrative case examples to illustrate successful applications across distinct domains. These include clinical decision support (Pediatric Emergency Care Applied Research Network [PECARN] head trauma and abdominal trauma rules), predictive modeling (neonatal sepsis detection and bronchopulmonary dysplasia [BPD] prediction), and automated screening (autism spectrum disorder [ASD]). These cases were chosen to represent diverse pediatric age groups, clinical contexts, and informatics applications.

**Figure 1.**
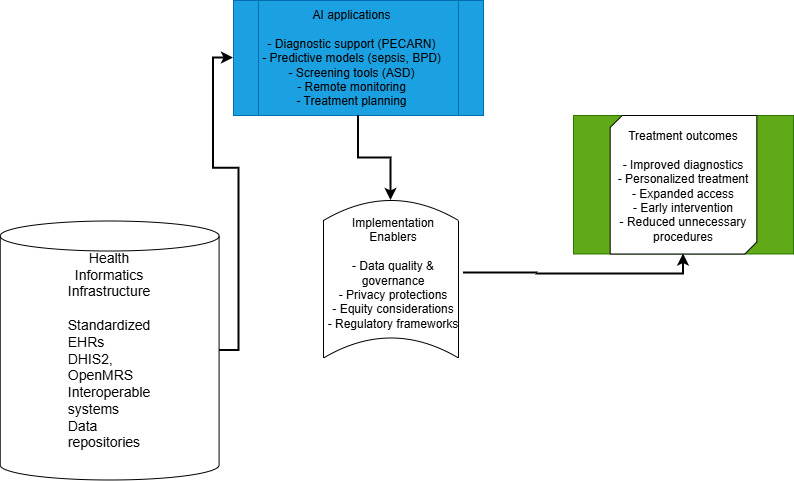
Conceptual framework for AI-enhanced pediatric care delivery. This framework illustrates the pathway from health informatics infrastructure through AI applications to improved pediatric care outcomes as explored in this viewpoint. The arrows represent conceptual relationships. AI: artificial intelligence; ASD: autism spectrum disorder; BPD: bronchopulmonary dysplasia; DHIS2: District Health Information Software 2; EHR: electronic health record; OpenMRS: Open Medical Record System; PECARN: Pediatric Emergency Care Applied Research Network.

This methodology had several limitations. This synthesis may preferentially reflect selection bias toward well-documented implementations in high-resource settings. Emerging applications and ongoing trials may not be fully captured. The evidence base for implementation in LMICs remains limited, representing an important research gap highlighted in this viewpoint. Textbox 1 summarizes the key potential benefits and implementation challenges of AI and health informatics in pediatric care, organized by domain. Figure 1 presents the conceptual framework informing this viewpoint, illustrating conceptual linkages from health informatics infrastructure through AI applications to pediatric care outcomes.

Textbox 1. **High-level synthesis of key benefits and implementation challenges of artificial intelligence and health informatics in pediatric care. Benefits reflect emerging evidence from selected implementations and should be interpreted cautiously. Challenges highlight issues that require addressing.**

Potential benefits

- Diagnostic support—artificial intelligence (AI) tools may improve diagnostic accuracy for conditions such as pneumonia
- Predictive modeling—AI models show promise for early detection of high-risk conditions, including neonatal sepsis and bronchopulmonary dysplasia
- Personalized treatment—AI-supported clinical decision tools may inform individualized treatment selection
- Accessibility—telemedicine platforms integrated with AI may expand access to pediatric specialists in underserved areas
- Efficiency—automated screening tools and remote monitoring systems enable earlier intervention
- Remote monitoring—mobile health platforms with AI analytics may support chronic disease management

Implementation challenges

- Data quality—limited availability of high-quality, representative pediatric data sets
- System integration—incompatibility between legacy health information systems and newer AI technologies
- Algorithmic bias—risk of perpetuating health inequities when models are trained on unrepresentative data
- Privacy and ethics—need for robust privacy protections and informed consent frameworks
- Regulatory variation—limited pediatric-specific AI governance
- Socioeconomic disparities—unequal distribution of AI implementations across income levels

## Population-Level Benefits of Informatics and AI in Pediatric Health

Health informatics and AI offer several potential benefits that would improve global pediatrics. These technologies may improve accuracy, personalization of pediatric care, accessibility, early detection and intervention, and support for pediatric mental health services. One such benefit is efficiency and accuracy. Algorithm-based diagnostic tools have demonstrated effectiveness in selected clinical contexts. For instance, convolutional neural networks have been applied to pediatric medical imaging, including the diagnosis of leukemia [6], as well as other conditions such as pneumonia and retinopathy of prematurity [7]. Systematic reviews suggest that some AI systems have performed comparably to human experts in interpreting radiographs and computed tomography (CT) images [8], while validation studies in ophthalmic imaging have demonstrated expert-level performance in specific diagnostic tasks [9]. This evidence suggests that AI can enhance aspects of pediatric care delivery in specific contexts. Beyond diagnostic imaging, AI-driven systems have been explored for continuous monitoring and personalized management in pediatric chronic conditions, including the development of knowledge-enabled conversational systems to support asthma self-management by integrating patient-reported data, environmental information, and domain knowledge to deliver contextualized and personalized guidance [10]. In more acute settings, ML approaches have been applied to pediatric critical care EHR data to identify prognostic clusters and clinically relevant patterns, highlighting the potential of data-driven models in critically ill pediatric populations [11].

Another notable benefit is personalization. Health informatics and AI support the development of targeted treatments. AI analyzes patient characteristics to inform treatment decisions for different pediatric patients. This is an evolution from the usual use of a definite general scheme of therapy. ML algorithms play important roles in such personalization. In pediatric oncology, AI-based decision-making tools are increasingly being developed to support clinicians in selecting a chemotherapy regimen based on tumor biomarkers and patient characteristics [12]. By enabling more precise treatment selection, these tools may reduce adverse effects. AI-driven conversational systems, such as the chatbot Tess, have been implemented to provide adolescents managing chronic conditions with tailored information and emotional support [13]. Digital tools, therefore, show promise in addressing the psychosocial needs of children and adolescents with chronic illnesses.

There is also the benefit of accessibility. Ensuring equitable access to high-quality services remains a persistent challenge in resource-constrained settings, where limitations in infrastructure, workforce capacity, and system-level quality standards affect service delivery [14]. Telemedicine platforms supported by health informatics technology play an important role in improving accessibility. By incorporating NLP and real-time analyses, these platforms enable patients, especially children in underserved areas, to seek consultations from specialists. AI-powered translation and NLP tools have been explored to address communication and language barriers in global health settings, which may be particularly relevant for pediatric populations in multilingual and resource-constrained contexts [15].

The benefit of early detection and intervention cannot be overlooked. Health informatics and AI applications in pediatrics extend to opportunities for early detection and intervention. For instance, AI-enhanced imaging modalities in pediatric cardiology, including echocardiography and other cardiac imaging techniques, have demonstrated potential to improve diagnostic insight and support timely detection of congenital heart disease [16]. For chest radiography, AI applications in tuberculosis and pneumonia diagnosis have demonstrated promising diagnostic performance in pediatric populations, with potential to support clinical decision-making across diverse care settings [8]. Apart from diagnostics, health informatics and AI expand the availability of preventive care. For example, health informatics and AI have been used to predict sepsis and BPD in neonates in the neonatal intensive care unit [17], while other ML approaches have explored early identification of neonatal sepsis [18]. Such predictive models may support earlier clinical intervention when interpreted within established care pathways.

## Critical Evidence: Real-World AI Applications Transforming Pediatric Care

Health informatics and AI are not merely concepts in pediatric health care, as these technologies have demonstrated measurable outcomes in clinical settings. The following case studies illustrate applications across 3 domains: clinical decision support, predictive modeling, and automated screening. In this viewpoint, these domains are selected to represent diverse pediatric contexts and informatics approaches, encompassing the PECARN rules, AI in neonatal sepsis, and AI diagnosis of ASD.

### PECARN Clinical Decision Rules

The PECARN clinical decision rules represent a large-scale application of evidence-based clinical prediction modeling in pediatric emergency medicine rather than a traditional AI-driven system. PECARN is a coordinated network of pediatric emergency departments across the United States and Canada. Researchers derived and prospectively validated statistical prediction rules to identify children at very low risk of clinically important traumatic brain injury. These predictors were incorporated into a validated clinical decision support rule that assists physicians in identifying children at very low risk for clinically important traumatic brain injury, thereby helping determine when CT imaging can be safely avoided [19]. As one of the earliest large-scale validated prediction frameworks in pediatric emergency medicine, PECARN laid important groundwork for subsequent AI-enabled decision support systems.

A large multicenter prospective validation study involving 7542 children with blunt abdominal trauma and 19,999 children with minor head trauma demonstrated high diagnostic accuracy of the PECARN prediction rules. The intra-abdominal injury rule showed 100% sensitivity for identifying children requiring acute intervention. For traumatic brain injury, the rule demonstrated 100% sensitivity in children younger than 2 years and 98.8% sensitivity in children aged 2 years and older for clinically important traumatic brain injuries. Implementation of these validated clinical prediction rules supports safer decision-making regarding CT imaging and may reduce unnecessary radiation exposure while maintaining high-quality care [19]. This example demonstrates how rigorously validated, data-driven clinical decision support tools, when embedded within structured informatics frameworks, can improve diagnostic accuracy while reducing unnecessary radiation exposure.

### ML for Neonatal Condition Prediction

Neonatal sepsis continues to be among the most morbid and deadly illnesses in infants, especially in LMICs. A global systematic review and meta-analysis reported an estimated incidence of 2824 cases per 100,000 live births, with a mortality rate of 17.6%, underscoring the persistent burden of disease worldwide [20]. Early identification is especially challenging in the neonatal intensive care unit, where clinical presentations are often nonspecific and may delay diagnosis and treatment.

Early sepsis identification is critical, yet clinical presentations are often nonspecific. This leads to delayed diagnosis and treatment. One model was trained on large vital sign datasets, including heart rate variability, respiration, and oxygen saturation from high-risk neonates [18]. Such approaches may support earlier clinical intervention and improve outcomes in vulnerable neonatal populations.

Similarly, predictive modeling has been applied to BPD risk assessment in preterm infants. The study showed that using AI in predicting BPD in preterm infants achieved 88% sensitivity and 91% specificity. The study also enrolled 61 preterm infants (gestational age 24‐31 wk) and identified the 26 (43%) infants who developed BPD. When using clinical data alone (birth weight, gestational age, and surfactant treatment), the model showed 74% sensitivity and 82% specificity. Performance improved to 88% sensitivity and 91% specificity when combined with AI-analyzed spectral data from physiological signals [18]. These findings suggest that ML-enhanced analysis of continuous physiologic data may improve early risk stratification in high-risk neonates.

### ML for Autism Screening

ASD is a neurodevelopmental condition characterized by differences in social communication and restricted or repetitive behaviors [21]. Early ASD identification and referral to intervention programs are associated with improved developmental outcomes. However, comprehensive diagnostic assessments for ASD require specialized clinical expertise that may be limited in many developing countries. The traditional Autism Diagnostic Observation Schedule-Generic (ADOS) assessment takes between 30 and 60 minutes to administer. Families may wait as long as 13 months between initial screening and diagnosis. ML approaches have been explored to streamline these assessment challenges.

Wall and colleagues at Harvard Medical School developed an AI-based screening tool, which analyzes data from behavioral and cognitive assessments to identify patterns of ASD. Their study analyzed 612 individuals with autism using existing ADOS datasets, alongside comparison groups from independent cohorts. These included the Autism Genetic Resource Exchange and the Boston Autism Consortium. The research tested 16 different ML algorithms to identify the most effective approach for autism classification. The resulting classifier used only 8 items from the original 29-item ADOS module. This represented a 72.4% reduction in assessment items. This approach achieved an accuracy of 100% sensitivity and 100% specificity in the initial training set [22]. It correctly classified all 612 individuals with autism and all 15 nonspectrum individuals. When tested on independent datasets, the classifier maintained performance with 99.7% accuracy on the Simons Simplex Collection and 100% accuracy on the Boston Autism Consortium dataset [18,22]. These results are promising, but the small sample sizes and need for validation in diverse clinical populations limit generalizability.

## Current Applications

Health informatics provides the foundational infrastructure for integrating AI into clinical workflows. Standardized health information systems enable mechanisms for diagnostics, treatment planning, decision support, and patient monitoring. Examples of diagnostic innovations in health care include molecular detection platforms such as M gene–targeted quantitative reverse transcription polymerase chain reaction assays for pathogen identification [23]. The success of health informatics–enabled AI implementations in this area informs the potential for leveraging ML and AI to improve pediatric care. In diagnostic applications, standardized EHR systems organize clinical data that ML algorithms can analyze to support diagnostic decisions. Several studies report that ML systems demonstrate promising diagnostic performance for detecting pulmonary conditions in pediatric chest radiographs [6,8]. Similarly, AI-assisted histopathology has been explored for detecting medulloblastoma. A study by Attallah reported improved diagnostic accuracy for tumor subtyping compared to conventional methods [24]. These AI-supported tools may help reduce diagnostic errors in pediatric practice.

Applications in treatment personalization integrate EHR data with ML to support individualized treatment decisions. Clinical data from EHRs, including genetic and clinical data and patient environmental factors, can inform ML models to predict treatment response. Pediatric oncology has been a particular focus for such applications, where AI supports clinicians in selecting chemotherapy regimens based on the tumor characteristics and patient factors [12]. AI applications have also been explored in surgical planning, where patient-specific anatomic models may assist pediatric surgeons in virtual surgical planning, potentially improving procedural accuracy.

Remote monitoring applications leverage mobile health platforms and wearable devices for pediatric chronic disease management. These systems collect and analyze physiological data from wearables and EHRs for continuous vital sign monitoring among children with chronic conditions. Real-time alerts may support early interventions that could potentially reduce hospital readmission rates [25]. Studies suggest that ML applied to pediatric critical care data may provide prognostic insights and support risk stratification in hospitalized children [11].

## Challenges in AI-Enabled Pediatric Systems

Implementation of AI in health care, including pediatric health informatics, faces several interconnected challenges. These challenges include technical challenges related to data quality, system integration, and ethical and legal concerns regarding privacy and transparency, and socioeconomic disparities in access and resources [26]. Technical challenges in pediatric data collection include small sample sizes, ethical constraints on research involving children, and developmental heterogeneity across the pediatric population [27]. AI models trained on historical data may be limited by data that are inadequate, contradictory, or biased. Training datasets often lack adequate representation of diverse populations, including racial and other demographic groups, potentially leading to biased model outputs [28]. High-quality pediatric data from LMICs remains limited due to health system infrastructure gaps and fragmented health information systems [29]. Addressing these gaps requires investment in strong data-sharing platforms and strengthened digital infrastructure to enable representative pediatric data collection globally.

System integration challenges may arise from differences in data structures, data quality, and governance frameworks between existing health information systems and newer AI technologies [30]. Interoperability standards such as Health Level Seven Fast Healthcare Interoperability Resources are being adopted to address these technical barriers, though implementation remains inconsistent globally [31].

There is also a challenge of algorithmic bias that poses a significant problem when deploying health informatics and AI in pediatric care. Models trained on biased datasets may perpetuate existing health inequalities. For example, algorithms developed using data collected in high-income countries may perform poorly in LMICs because epidemiologic profiles and models of health care delivery are different [32,33]. Bias may also arise from subjective labeling of training data or underrepresentation of minority populations in data collection.

Ethical and legal concerns include data privacy protections, regulatory variations, and algorithmic transparency that affect trust in AI systems. Pediatric health data are particularly sensitive, as privacy breaches may have long-term implications for individuals [30]. Most jurisdictions have data protection frameworks. For example, the General Data Protection Regulation in Europe and the Health Insurance Portability and Accountability Act in the United States [34]. Beyond privacy, algorithmic transparency presents major ethical challenges. A review of AI tools in LMIC health care settings noted that many systems relied on complex or “black-box” algorithms with limited interpretability, raising concerns about transparency and clinical trust [32].

Technology changes faster than regulatory policies. The regulatory framework in pediatrics reflects this dynamic. Some developed nations have advanced AI governance frameworks. For example, Singapore has developed a Model AI Governance Framework. Other nations, such as the United Kingdom and the United States, are continuously developing provisions to regulate AI. Many LMICs appear to be in the earlier stages of developing robust regulatory frameworks for AI in health care [30]. International organizations, including the WHO and International Telecommunication Union, are working to establish global standards and guidance for responsible AI deployment in health [35].

Socioeconomic disparities in AI adoption for pediatric health reflect broader global health inequities. A review by Ciecierski-Holmes et al [32] found that 80% of AI implementations were in upper-middle-income countries. There were only 10% in low-income countries and 10% in lower-middle-income countries. These findings reflect the uneven distribution of AI health care technologies globally. Multiple factors contribute to these disparities, including limited digital infrastructure, a shortage of technical expertise, competing health system priorities, and insufficient investment in health information systems [35]. Advancing equitable AI adoption requires addressing these structural barriers alongside technology development.

## Solutions for Scalable Pediatric AI Adoption

### Enhanced Data Quality and System Integration

Improving data quality and availability is a critical priority. This is significant because only 50% of AI health care applications in LMICs documented their training datasets adequately [32]. ML performance depends on the quality and representativeness of training data. Given challenges in pediatric research data collection, the successful adoption of AI in pediatrics would largely benefit from collaborative efforts to develop a uniform data source. Such a source would hold pediatric practice data from diverse geographic and demographic contexts. This would create uniform data repositories that provide adequate and high-quality data for the development of health information systems and AI models in pediatric care.

System integration approaches should build on existing health information infrastructure rather than requiring wholesale replacement. For this integration to work, it is recommended that open-source platforms be used for compatibility. As an example, existing EHRs would be a great addition to AI systems adopted in pediatric health [36]. Some widely adopted open-source health information systems include District Health Information Software 2 for health management information, Open Medical Record System for electronic medical records, and Bahmni for hospital management. These platforms provide foundational platforms that can integrate AI applications while maintaining local adaptability. Such open-source platforms can facilitate collaborative development and promote application programming interface interoperability across diverse geographical contexts.

### Privacy, Security, and Equity

Data privacy and security are fundamental to ethical health informatics and AI deployment in pediatric care. Some legal protections relating to the protection of personal information include the General Data Protection Regulation in Europe and the Health Insurance Portability and Accountability Act in the United States. However, many countries have distinct legal traditions and frameworks rooted in international human rights principles. Some of these include the right to privacy established in post–World War II conventions, such as the Universal Declaration of Human Rights and the Nuremberg Code’s protections for research participants (Universal Declaration of Human Rights/Nuremberg Code). Such regulations and policies provide an important legal foundation for protecting personal information [37]. Technical measures that can be implemented include encryption for data at rest and in transit, role-based access controls, and audit trails. Privacy-enhancing technologies, such as federated learning and anonymization, enable collaborative model development without violating the patient’s privacy [30]. Privacy protections must be complemented by informed consent processes that respect individual and community autonomy, including the right to opt out of data use for AI development. Health care organizations should set up independent ethics committees to monitor data protection and compliance with ethics rules.

Bridging the digital divide is critical to ensuring equitable access to health informatics and AI-driven pediatric care. Strategic investments in digital infrastructure, such as connectivity, computing resources, and technical capacity, are essential for wider adoption in underserved regions [24]. Mobile health platforms integrated with AI applications may offer scalable options for delivering health care services to areas with limited resource access. These platforms can support diagnostic and consultation services, potentially reducing the need for patients to travel to higher-level health care facilities [15]. Co-design approaches that engage local communities and health workers will bolster cultural appropriateness and language accessibility in pediatric practice.

### Cost-Effective Implementation Strategies

Resource constraints present barriers to AI adoption in many settings. Hiring specialized experts during the initial phase of implementation of health informatics and AI, and the cost of training employees to acquire the new skills required for performing artificially intelligent tasks requires substantial resources [32]. Public-private partnerships may facilitate implementation in some contexts, but careful attention to governance, sustainability, and public interest protections is essential. Adopting open-source platforms may help reduce licensing costs and support transparent, collaborative development of AI tools, particularly in resource-constrained settings [30]. Another important measure is prioritizing AI applications to address high-burden conditions. For example, AI applications used in neonatal sepsis, pneumonia, and malnutrition would ensure resources are dedicated to areas with the greatest health impact.

## Conclusions

This viewpoint has examined the intersection of health informatics and AI in pediatric health care. Using existing case studies and literature, we identified the current applications, benefits, and challenges of using health informatics and AI in pediatric health care. These technologies show promise in enhancing diagnostic accuracy, expanding access to care, and supporting more personalized treatment approaches in pediatric settings.

Significant implementation barriers persist, including challenges related to data quality, technical implementation, ethical governance, and persistent socioeconomic disparities that disproportionately affect LMICs. Addressing these challenges through collaborative, equity-driven efforts is essential to realizing AI’s potential in enhancing global pediatric care. The tools exist, the need is urgent, and the time for global action is now.

Future research should explore long-term outcomes of AI implementations in diverse pediatric settings, develop context-appropriate validation frameworks, and establish governance structures that ensure ethical and equitable deployment.

## Abbreviations

ADOS: Autism Diagnostic Observation Schedule
AI: artificial intelligence
ASD: autism spectrum disorder
BPD: bronchopulmonary dysplasia
CT: computed tomography
EHR: electronic health record
LMICs: low- and middle-income countries
ML: machine learning
NLP: natural language processing
PECARN: Pediatric Emergency Care Applied Research Network
WHO: World Health Organization
